# Taurine transporter SLC6A6 expression promotes mesenchymal stromal cell function

**DOI:** 10.1038/s41419-025-08233-4

**Published:** 2026-01-08

**Authors:** Christina M. Kaszuba, Benjamin J. Rodems, Sonali Sharma, Cameron D. Baker, Edgardo I. Franco, Takashi Ito, Palomi Schacht, Kyle P. Jerreld, Emily A. Johnson, Bradley R. Smith, Chen Yu, Emily R. Quarato, Francisco A. Chaves, Jane L. Liesveld, Laura M. Calvi, Hani A. Awad, Roman A. Eliseev, Jeevisha Bajaj

**Affiliations:** 1 Department of Biomedical Engineering, University of Rochester, Rochester, NY USA; 2 Wilmot Cancer Institute, University of Rochester Medical Center, Rochester, NY USA; 3 Department of Biomedical Genetics, University of Rochester Medical Center, Rochester, NY USA; 4 Genomics Research Center, University of Rochester Medical Center, Rochester, NY USA; 5 Department of Bioscience and Technology, Graduate School of Bioscience and Technology, Fukui Prefectural University, Fukui, Japan; 6 Department of Biology, University of Rochester, Rochester, NY USA; 7 Center for Musculoskeletal Research, University of Rochester, Rochester, NY USA; 8 Department of Orthopaedics, University of Rochester Medical Center, Rochester, NY USA; 9 Department of Biochemistry and Biophysics, University of Rochester Medical Center, Rochester, NY USA; 10 Department of Medicine, University of Rochester Medical Center, Rochester, NY USA; 11 Department of Pharmacology and Physiology, University of Rochester Medical Center, Rochester, NY USA

**Keywords:** Cell biology, Senescence

## Abstract

Mesenchymal stromal cell (MSC) differentiation is critical for the development, maintenance, and repair of bone tissue. MSCs also play a key role in regulating self-renewal and differentiation of normal hematopoietic and leukemic stem cells. Our prior work has identified a key role of taurine produced by bone marrow osteolineage cells in supporting the growth of taurine transporter (TauT or Slc6a6) expressing leukemia cells. Here, we analyze multiple murine non-hematopoietic bone marrow single-cell RNA-sequencing datasets and discover that TauT expression is enriched in MSCs in vivo. Although taurine supplements have been shown to mitigate bone defects in aged mice, its role in regulating MSC populations that give rise to bone cells is poorly understood. Using TauT genetic loss-of-function murine models, we find that TauT loss impacts murine MSC populations in vivo and impairs MSC osteogenic differentiation in vitro. This is associated with decreased bone mineral density and bone strength in young and aged TauT knockout mice. Importantly, shRNA-based knockdown of TAUT expression in primary human donor MSCs reduces osteogenic differentiation. TauT null MSCs are unable to support self-renewal and expansion of co-cultured hematopoietic stem and progenitor populations, indicating broad functional defects. Mechanistically, TauT loss results in downregulation of inositol metabolism, increased oxidative stress, and reduced Wnt/β-catenin signaling, which induce MSC senescence. Collectively, our data identifies taurine as a key regulator of MSC maintenance and osteogenic fate determination.

## Introduction

The bone marrow microenvironment (BMME) is a complex system consisting of extracellular matrix, as well as multiple cell types including endothelial cells, mesenchymal stromal cells (MSCs), osteoblasts, fibroblasts, and hematopoietic populations. The BMME plays a critical role in providing mechanical and structural support to bone, promoting self-renewal of hematopoietic stem cells, and regulating normal and malignant hematopoietic cell growth [1–5].

MSCs are key components of the BMME that can differentiate along osteolineage and adipolineage, providing essential support for bone function [6]. MSC osteo-differentiation is stimulated by growth factors including transforming growth factor-beta, bone morphogenic proteins, fibroblast growth factor; transcription factors such as Osterix and Runx2; and signaling pathways like Hedgehog, Notch, and Wingless/integrated (Wnt) [7–11]. Aberrant MSC differentiation has been linked to bone disorders such as osteoporosis, osteopenia, and osteogenesis imperfecta [12, 13]. Given their key role in multiple physiological processes, defining new factors that promote MSC fitness may help inform therapies promoting bone homeostasis and hematological support.

Metabolic regulators within the BMME are known to be essential for bone homeostasis, in part by their regulation of MSCs. For instance, glutamine is critical for MSC differentiation, and necessary to maintain bone mineral density [14, 15]. Arginine and lysine are key components of collagen, which are critical for bone formation and structure [14, 16]. The non-essential amino acid taurine can inhibit age related bone loss in 24-month-old mice, indicating that taurine may promote bone health in the context of aging [17]. However, it is not well established if taurine is also essential for bone development and maturation in young populations.

Taurine is required for multiple physiological functions such as osmoregulation, energy metabolism, and antioxidant defense [4, 18, 19]. These functions rely on intracellular taurine levels, which are primarily controlled by the expression of the taurine transporter, TauT. TauT, encoded by the SLC6A6 gene, is the primary, high affinity sodium chloride-dependent transporter of taurine. Our earlier work has shown that taurine produced by osteolineage cells can support leukemia growth. Further, osteoblast and osteocyte in vitro cultures grown in the presence of taurine show increased proliferation and reduced cell death [20, 21]. However, the role of taurine in MSC maintenance and differentiation remains poorly understood. This is of particular interest in the context of young populations where MSCs are critical for bone development and homeostasis [22].

Here, using young (16 week old) and middle aged (40 week old), *Slc6a6* knockout mice, we identify a key role of taurine uptake by MSCs in maintaining their ability to differentiate along the osteolineage, support hematopoietic populations, and promote bone homeostasis.

## Results

### Mesenchymal stromal cells in the bone marrow express high levels of Slc6a6

**Fig. 1:**
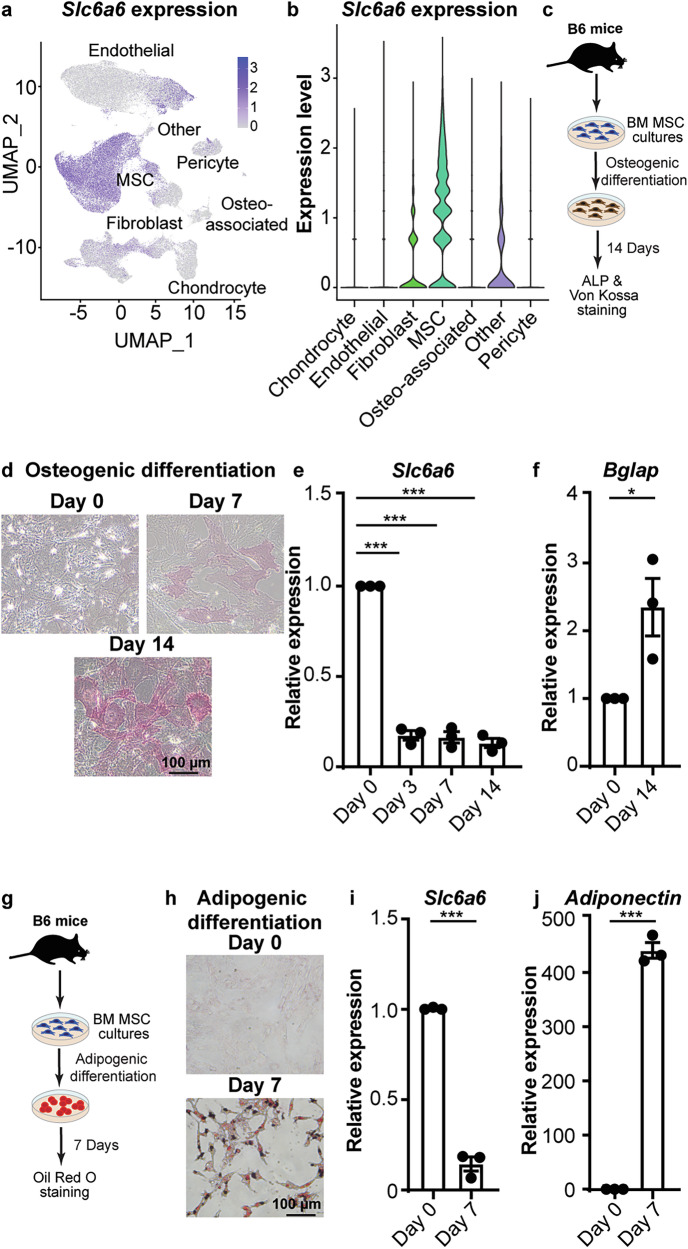
Slc6a6 expression in non-immune bone marrow populations. **a** Slc6a6 expression in 7 distinct non-hematopoietic bone and bone marrow stromal cell clusters (scale bar represents expression level). **b** Violin plot of Slc6a6 expression in non-hematopoietic cells (Data from GSE226644, GSE108892, GSE122467, GSE128423). **c** Experimental strategy for MSC osteogenic differentiation. **d–f** Microscopy images of alkaline phosphatase and von Kossa staining (**d**), Slc6a6 expression (**e**), and Bglap expression (**f**) in MSCs undergoing osteogenic differentiation (**e**, **f**, mean ± s.e.m.; data are combined from two-three independent experiments; **e** one-way ANOVA). **g** Experimental strategy for MSC adipogenic differentiation. **h–j** Oil Red O staining (**h**), Slc6a6 expression (**i**), and Adiponectin expression (**j**) in MSCs undergoing adipogenic differentiation (**i**, **j**, mean ± s.e.m.; data are combined from two-three independent experiments) (*p < 0.05, **p < 0.01. ***p < 0.001). All analyses are from unpaired two-tailed Student’s t-test or as indicated.

Taurine levels can decline with age in some human populations, and taurine supplements can mitigate the onset of bone defects in aged mice [17, 23]. To determine the cell types that can take up taurine, we analyzed Slc6a6 expression in single-cell RNA-sequencing (scRNA-seq) datasets of non-immune murine bone and bone marrow populations from 6-22 week old mice [4, 24–26]. Our analysis indicates that while all MSCs and a small subset of endothelial cells express high levels of Slc6a6 (Fig. 1a, b, and S1a), it is not detectable in cells differentiating along the osteolineage pathway. Importantly, we do not detect expression of other non-specific, low-efficiency taurine transporters, e.g. Slc6a13, Slc16a6, Slc36a1 [27–29], across all analyzed cell types (Fig. S1b). These data indicate that the impact of taurine uptake by Slc6a6 on bone homeostasis may be driven by its role in MSCs.

To validate Slc6a6 expression in cultured MSCs, we isolated murine MSCs and tested their differentiation ability [4, 30, 31]. Our assays showed that these MSC cultures have high expression of CD29 (100%), CD44 (99%), and CD105 ( ~ 45–49%) (Fig. S1c–e), confirming their purity [4, 30, 31]. Consistent with our scRNA-seq analysis, we find that MSCs rapidly downregulate Slc6a6 expression during osteogenic and adipogenic differentiation (Fig. 1c–j). These data indicate that Slc6a6 is primarily expressed in undifferentiated MSCs. We thus focused our studies on MSCs as they are known to be critical for bone development and maintenance.

### Effect of TauT loss on MSC and endothelial populations in vivo

**Fig. 2:**
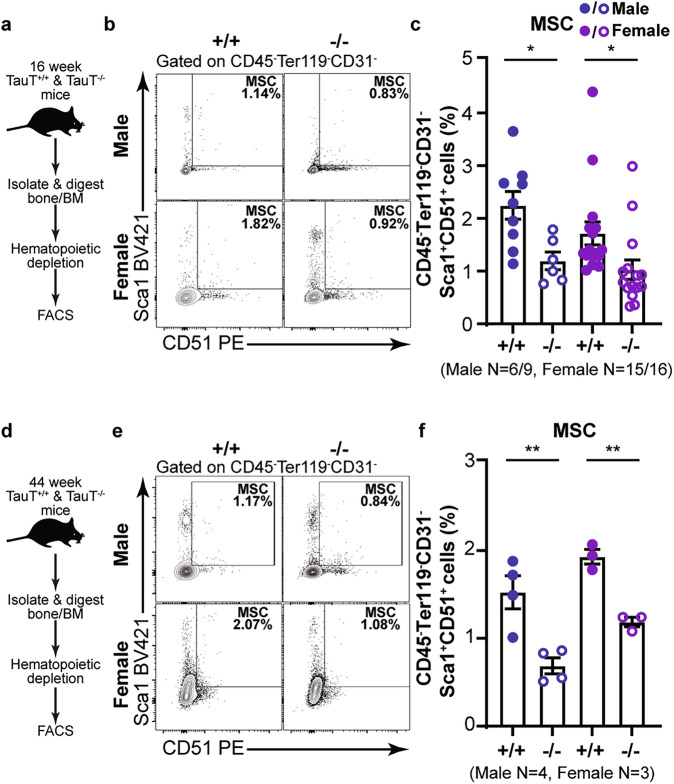
TauT loss impacts MSCs in vivo. **a** Experimental strategy used to analyze microenvironmental cells in 16 week TauT^+/+^ ( + /+) and TauT^−/−^ (−/−) mice. **b**, **c** Representative FACS plots (**b**) and quantification of MSCs (**c**) in bone marrow stroma (mean ± s.e.m.; data are combined from seven independent experiments). **d** Experimental strategy used to analyze microenvironmental cells in 40 week TauT^+/+^ ( + /+) and TauT^−/−^ (−/−) mice. **e**, **f** Representative FACS plots (**e**) and quantification of MSCs (**f**) in bone marrow stroma (mean ± s.e.m.; data are combined from three independent experiments). (*p < 0.05, **p < 0.01. ***p < 0.001). All analyses are from unpaired two-tailed Student’s t-test.

High expression of Slc6a6 (TauT) in MSCs and some endothelial populations indicates that taurine uptake may be essential for sustaining these populations. To test this, we used a global TauT knockout murine model [4, 32]. While TauT knockout mice are born in Mendelian ratios, they can develop age dependent impairments in the retina [33] and bone [17]. To determine if TauT loss impacts BMME populations in vivo, we analyzed stromal populations in the femur of young adult (16 week) TauT^+/+^ and TauT^−/−^ mice (Fig. 2a). Our experiments showed that TauT^−/−^ mice have a 40–47% lower frequency of CD45^-^TER119^-^CD31^-^CD51^+^SCA1^+^ MSCs relative to wild-type controls (Fig. 2b, c). While there was a striking loss in MSC frequency, we did not detect any change in either arteriolar or sinusoidal endothelial cells (Fig. S2a–c). These MSCs also expressed high levels of other reported MSC markers such as CD29 (100%), CD44 (90-95%), and CD105 ( ~ 82%) [30, 31] (Fig. S2d–f), confirming their identity.

To determine if alterations in BMME populations are also seen in the context of aging, we analyzed these populations in 40 week old TauT^+/+^and TauT^−/−^ mice (Fig. 2d). In addition to a 39–55% decrease in MSC frequency with TauT loss (Fig. 2e, f), we noted a trending decline in arteriolar and sinusoidal endothelial cells (Fig. S2g–i). Our analysis of publicly available datasets of skeletal stem/progenitor cells from young and aged human fractured bones (13–94 years old) show that SLC6A6 expression decreases with age (Fig. S2j) [34]. This suggests that loss of SLC6A6 expression in MSCs may contribute to bone defects seen in the context of aging.

Collectively, these data indicate that while TauT expression is essential for sustaining MSC populations, it may be dispensable for maintaining endothelial subsets in the bone marrow at steady-state.

### TauT loss impairs MSC function

**Fig. 3:**
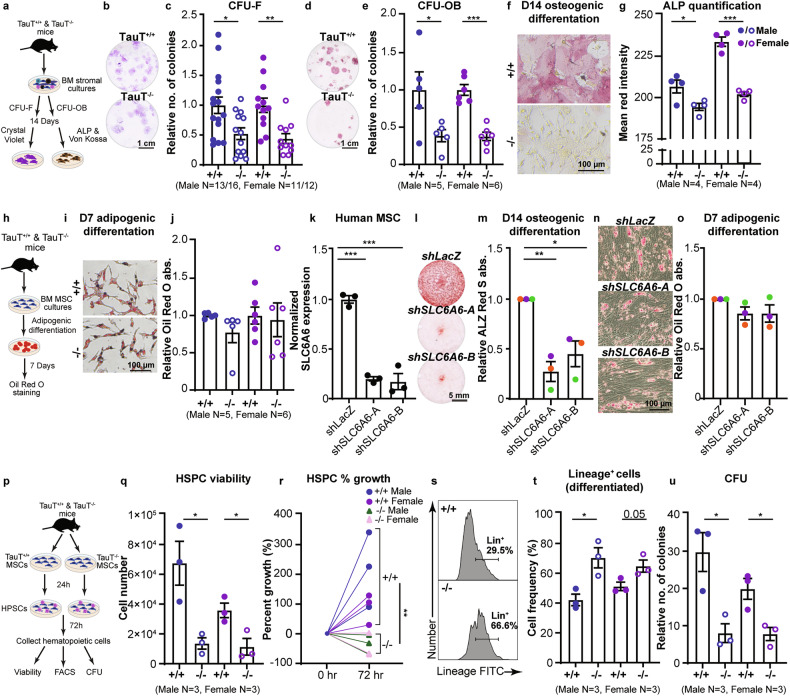
The effect of TauT loss on MSC function in vitro. **a** Experimental strategy used for colony forming unit fibroblast (CFU-F) and osteoblast (CFU-OB) assays from bone marrow cells from TauT^+/+^ ( + /+) and TauT^−/−^ (−/−) mice. **b**, **c** Crystal violet staining (**b**) and CFU-F fold change (**c**) (mean ± s.e.m.; data are combined from six independent experiments). **d**, **e** Alkaline phosphatase (ALP) and von Kossa staining (**d**) and CFU-OB fold change (**e**) (mean ± s.e.m.; data are combined from six independent experiments). **f**, **g** Microscopy images of ALP and von Kossa staining (**f**) and quantification (**g**) in MSCs undergoing osteogenic differentiation for 14 days (mean ± s.e.m.; data are combined from three independent experiments). **h** Experimental strategy for MSC adipogenic differentiation. **i**, **j** Oil Red O staining (**i**) and relative absorbance quantification (**j**) in MSCs undergoing adipogenic differentiation (mean ± s.e.m.; data are combined from four independent experiments). **k** Normalized SLC6A6 expression in primary human donor bone marrow derived MSCs transduced with shRNAs targeting *LacZ* (control) or *SLC6A6* (mean ±s.d.; *n* = 3 technical replicates per cohort; one-way ANOVA). **l**, **m** Alizarin Red S staining (**l**) and quantification (**m**) in primary human donor bone marrow derived MSCs undergoing osteogenic differentiation for 14 days (mean ±s.e.m.; each color represents an independent human MSC sample; data are combined from three independent experiments; one-way ANOVA). **n**, **o** Day 7 Oil Red O staining (**n**) and absorbance quantification (**o**) in primary human bone marrow derived MSCs transduced with shRNAs targeting *LacZ* (control) or *SLC6A6* undergoing adipogenic differentiation (mean ±s.e.m.; data are combined from three independent experiments with three independent primary samples; one-way ANOVA). **p** Experimental strategy used for MSC and hematopoietic stem/progenitor cell (HSPC) coculture. **q** Trypan blue based cell viability of HSPCs 72 h post coculture with +/+ and −/− MSCs (mean ±s.e.m.; data are combined from five independent experiments). **r** Percent growth of HSPCs 72 hours post coculture with +/+ and −/− MSCs (mean ±s.e.m.; data are combined from five independent experiments; shape represents genotype). **s**, **t** Representative FACS histogram (**s**) and quantification (**t**) of Lineage^+^ frequency of HSPCs 72 hours post coculture with +/+ and −/− MSCs (mean ±s.e.m.; data are combined from five independent experiments). **u** CFU of HSPCs 72 h post coculture with +/+ and −/− MSCs (mean ±s.e.m.; data are combined from five independent experiments) (*p < 0.05, **p < 0.01. ***p < 0.001). All analyses are from unpaired two-tailed Student’s t-test or as indicated.

To determine if TauT expression in MSCs plays a functional role in these populations, we first determined the impact of TauT loss on MSC differentiation capacity (Fig. 3a). Our experiments identified a 48-56% decrease in fibroblast colony forming ability (CFU-F) (Fig. 3b, c) of bone marrow cells isolated from TauT^−/−^ mice compared to controls. In addition, TauT^−/−^ cells formed 48-56% fewer osteoblast colonies (CFU-OB) (Fig. 3d, e) compared to controls, indicating impaired osteogenic differentiation potential.

To confirm that MSCs, and no other cell types in the bone marrow, contribute to the observed CFU defects in TauT^−/−^ mice, we established purified bone marrow MSC cultures from TauT^+/+^ and TauT^−/−^ mice. We then tested their ability to undergo osteogenic and adipogenic differentiation over 7-14 days. While the TauT^−/−^ MSCs showed a small but significant decrease in alkaline phosphatase (ALP) and von Kossa-staining compared to controls (Fig. 3f, g), there was no effect on adipogenic differentiation (Fig. 3h–j).

To determine the significance of TauT expression in human cells, we established MSC cultures from three independent donors. These cultures expressed CD73 (100%), CD90 ( ~100%), and CD105 ( ~86%) (Fig. S3a–c), confirming purity [35]. We transduced these MSC cultures with either control (LacZ) or two independent shRNAs to knock down SLC6A6 expression by 5 to 5.8-fold (Fig. 3k), and then induced osteogenic differentiation. Consistent with our data from murine models, inhibiting SLC6A6 expression in human MSCs reduced their ability to undergo osteogenic differentiation by 55–73% as compared to controls (Fig. 3l, m), but did not impact their adipogenic differentiation potential (Fig. 3n, o).

These data suggest that while taurine uptake by MSCs is essential for establishing osteogenic fate, it may be dispensable for adipogenic potential.

### TauT loss impairs MSC’s ability to support hematopoietic stem and progenitor cells

In addition to bone formation, MSCs also support hematopoietic stem/progenitor cells (HSPCs) by secreting growth factors, chemokines, and cytokines [3, 36, 37]. We thus tested if TauT loss can impair the ability of MSCs to support HSPCs in vitro. To this end, we cocultured TauT^+/+^ and TauT^−/−^ MSCs with wild-type HSPCs (CD3ε^-^CD4^-^CD8^-^CD19^-^B220^-^CD11b^-^Ly6G^-^/Ly6C^-^Ter119^-^cKit^+^Sca1^+^; Fig. 3p). HSPCs cocultured with TauT^−/−^ MSCs had 3 to 5-fold reduced viability (Fig. 3q) and a 118% reduction in growth as compared to controls (Fig. 3r). This loss in growth and viability was accompanied by a 27–67% increase in frequency of Lin^+^ differentiated hematopoietic cells (Fig. 3s, t). In addition, the ability of cocultured HSPCs to form colonies in methylcellulose was reduced by ~3-fold (Fig. 3u). These data indicate that TauT^−/−^ MSCs have impaired ability to support HSPC expansion.

### Impact of TauT loss on bone properties

**Fig. 4:**
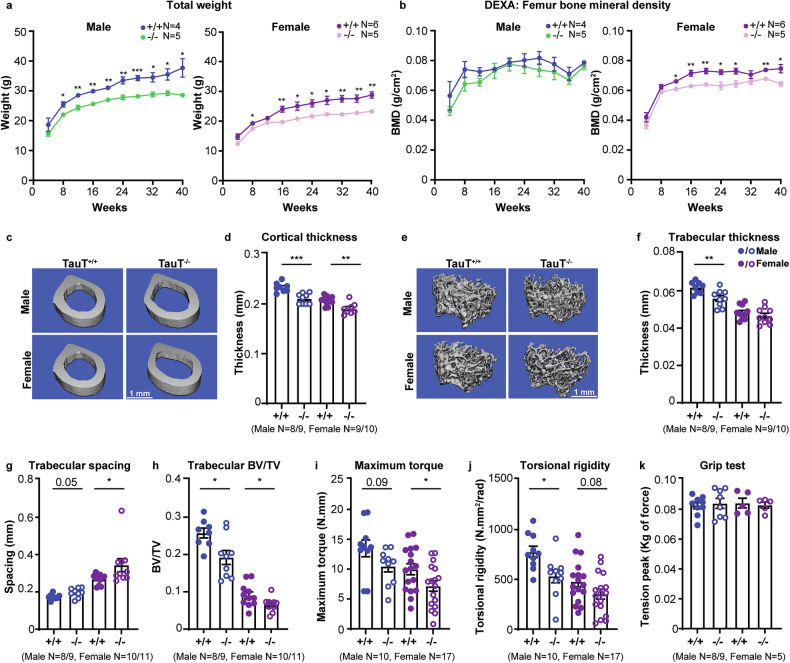
Physical and mechanical properties of bone in TauT^−/−^ mice. **a** Total weight of TauT^+/+^ ( + /+) and TauT^−/−^ (−/−) mice over a 40 week period (mean ±s.e.m.; data are combined from four independent experiments). **b** Dual-energy X-ray absorptiometry (DEXA) scans of femur bone mineral density (BMD) of +/+ and −/− mice over a 40 week period (mean ±s.e.m.; data are combined from four independent experiments). **c**, **d** Micro-CT pictographs of 16 week old mouse femur cortical region (**c**) and quantification of cortical thickness (**d**) (mean ±s.e.m.; data are combined from three independent experiments). **e–h** Micro-CT pictographs of 16 week old mouse femur trabecular region (**e**), quantification of trabecular thickness (**f**), trabecular spacing (**g**), and trabecular bone volume/total volume (BV/TV) (**h**) (mean ±s.e.m.; data are combined from three independent experiments). **i**, **j** Biomechanical torsion testing of 16 week old mouse +/+ and −/− tibia, quantification of maximum torque (**i**) and torsional rigidity (**j**) (mean ±s.e.m.; data are combined from three independent experiments). **k** Grip strength testing in 16 week old +/+ and −/− mice (mean ±s.e.m.; data are combined from five independent experiments) (*p < 0.05, **p < 0.01. ***p < 0.001). All analyses are from unpaired two-tailed Student’s t-test.

To directly test if impaired MSC function can impact bone properties, we first used dual-energy X-ray absorptiometry (DEXA) based scanning in young adult (16 week old) and middle-aged (40 week old) mice. Our experiments showed lower body weight of both 16 week old (14–17%) and 40 week old (19–24%) TauT^−/−^ mice as compared to controls (Fig. 4a). In addition, we noted a 12% decrease in femur bone mineral density (BMD) in female TauT^−/−^ mice beginning at 16 weeks, a time point consistent with adult characteristics [38] (Fig. 4b). While there was no change in femur fat percentage at 16 weeks, we noted a small decline in 40 week old TauT^−/−^ mice relative to controls (Fig. S4a), indicating that adipogenesis may be affected with age.

Our analysis of 16 week old TauT^−/−^ femur bone morphological structure using micro-computed tomography (micro-CT) revealed a 5–10% decrease in both cortical and trabecular thickness, a 11–21% increase in trabecular spacing, and a ~30% decrease in trabecular BV/TV as compared to controls, indicating an osteopenia-like phenotype [39] (Fig. 4c–h). Similarly, 40 week old TauT^−/−^ mice showed 18–30% lower cortical thickness and 10–17% reduced trabecular thickness as compared to controls (Fig. S4b–e). We noted no difference in tartrate-resistant acid phosphatase (TRAP) staining of osteoclasts in 16 and 40 week old TauT^−/−^ murine femurs as compared to controls (Fig. S4f–i) indicating that decreased physical bone properties with TauT loss likely reflect defects in MSC differentiation potential.

Our biomechanical torsion analysis showed that TauT^−/−^ tibias exhibit a 22–28% lower maximum torque and a 28-31% reduction in torsional rigidity as compared to controls (Fig. 4i, j). Since taurine is known to be important for muscle strength [40], we determined the impact of TauT loss on muscle function. We noted no differences in grip strength of 16 week old TauT^−/−^ mice as compared to controls (Fig. 4k). Moreover, our histological analysis on murine gastrocnemius muscles showed no changes in muscle fiber/nuclei structure or cross-sectional area (Fig. S4j, k), indicating that the observed defects in bone quality are not an indirect effect of altered muscle function in young adult mice.

Collectively, our data identify a decline in the in vivo bone physical and mechanical properties with TauT loss in young adult and middle-aged mice.

### TauT loss changes MSC metabolic profile

**Fig. 5:**
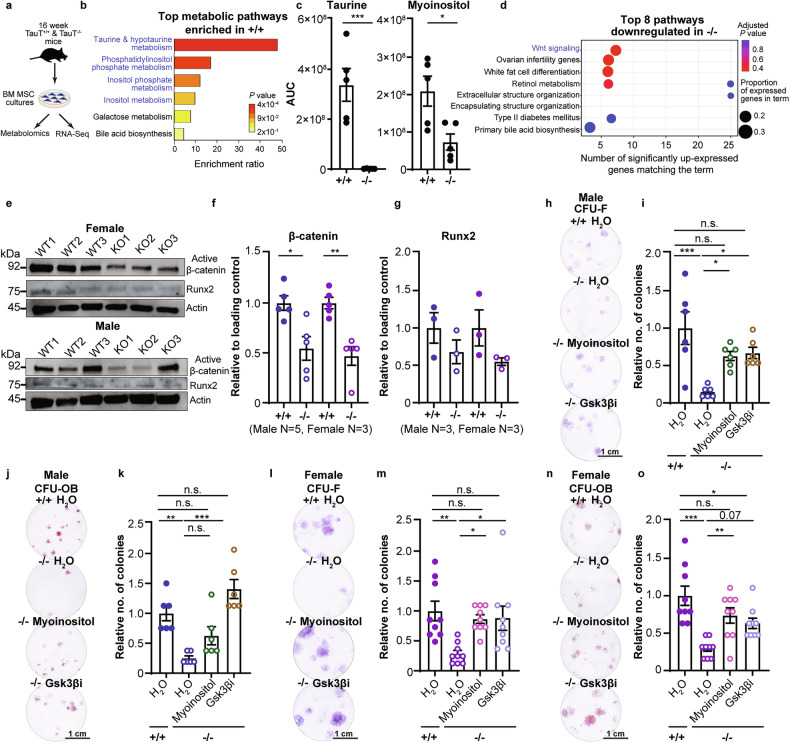
Metabolomic and transcriptomic analysis of MSCs in the absence of TauT. **a** Experimental strategy for untargeted metabolomics and bulk RNA-sequencing on 16 week old TauT^+/+^ ( + /+) and TauT^−/−^ (−/−) MSCs. **b** Unbiased Enrichr analysis (MetaboAnalyst; Padj ≤ 0.05) of top metabolic pathways enriched in +/+. **c** Quantification of taurine and myo-inositol (mean ±s.e.m.; data are combined from five independent MSC samples). AUC, area under the curve. **d** Gene sets significantly downregulated in −/− MSCs (*n* = 8 male and *n* = 7 female MSCs; blue text indicates pathways of interest). **e–g** immunoblot (**e**), and densitometric quantification (**f, g**) of indicated proteins (mean ±s.e.m. data are combined from four independent experiments). Original western blots are in Supplemental Fig. 7. **h**–**k** Crystal violet staining of CFU-F (**h**) and fold change (**i**) and alkaline phosphatase and von Kossa staining of CFU-OB (**j**) and fold change (**k**) of male +/+ and −/− MSCs treated with 1 mM myo-inositol or 5 µM CHIR99021 (Gsk3βi) (mean ±s.e.m.; n=three independent culture wells per mouse; one-way ANOVA). **l**–**o** CFU-F staining (**l**) and fold change (**m**) and CFU-OB staining (**n**) and fold change (**o**) of female +/+ and −/− MSCs treated with myo-inositol or CHIR99021 (mean ±s.e.m.; n=three independent culture wells per mouse; one-way ANOVA). All original western blots are in Supplemental Fig. 7. (*p < 0.05, **p < 0.01. ***p < 0.001). All analyses are from unpaired two-tailed Student’s t-test or as indicated.

Our prior work shows that taurine regulates the metabolic and transcriptomic profile of leukemia cells [4]. Thus, to determine the mechanisms underlying impaired MSC function in the absence of TauT, we carried out both untargeted metabolomics and RNA-sequencing analysis of TauT^+/+^ and TauT^−/−^ MSCs (Fig. 5a). Our LC/MS based assays identified downregulation of taurine metabolism and a profound 231-fold reduction in taurine levels with TauT loss (Fig. 5b, c), indicating that TauT mediated taurine uptake is the primary source of taurine in these cells. We also identified a significant downregulation of inositol metabolism and myo-inositol levels with TauT loss (Fig. 5b, c). In contrast, we noted an upregulation of arginine, glutathione, proline, and purine metabolism, as well as components of arginine metabolic pathway, such as l-ornithine, with TauT loss (Fig. S5a, b). These data indicate that TauT loss can lead to significant metabolic shifts in MSCs.

Our RNA-sequencing based gene-expression analysis of TauT^+/+^ and TauT^−/−^ MSCs identified 1139 downregulated and 951 upregulated genes in the absence of TauT (Padj<0.05). Upregulated genes in TauT^−/−^ MSCs primarily constituted pathways associated with inflammation and immune response, and included genes such as Bin3, Ccr1, and Slamf7, which are known to regulate pro-inflammatory responses (Fig. S5c). The downregulated genes in TauT^−/−^ MSCs primarily consisted of pathways associated with canonical Wnt signaling (Fig. 5d, S5d), consistent with the key role of this pathway in maintaining bone mass [10, 11]. In addition, we noted a 2.6 to 3.6-fold downregulation of growth factors such as stem cell factor (SCF), hepatocyte growth factor (HGF), and fibroblast growth factor (FGF) that are essential for HSPC support (Fig. S5e). This likely results in the impaired ability of TauT^−/−^ MSCs to support HSPCs growth and self-renewal (Fig. 3q–u).

Our experiments with TauT^−/−^ MSCs identified a 28–41% reduction in non-phosphorylated active β-catenin (Fig. 5e, f, Fig S5f), and 40% lower nuclear β-catenin (Fig. S5g) as compared to controls, confirming impaired Wnt pathway activation with TauT loss. Wnt/β-catenin signaling is known to promote MSC osteogenic differentiation by activating transcription of Runx2, a master regulator of osteoblast fate [41]. Consistent with this, we note a 32–45% reduction in Runx2 levels with TauT loss [42] (Fig. 5e, g).

To determine if myo-inositol and Wnt signaling play a functional role downstream of taurine in regulating osteogenic fate, we tested if ectopic activation of these signals can rescue defects seen with TauT loss. Our experiments showed that myo-inositol supplements can rescue both the fibroblast and osteoblast colony forming ability of TauT^−/−^ MSCs (Fig. 5h–o). Further, acute treatment with CHIR99021, a potent GSK3β inhibitor and Wnt/β-catenin pathway activator [43], could also rescue both fibroblast and osteoblast colony forming ability (Fig. 5h–o). These data indicate that taurine is essential for sustaining myo-inositol metabolism and Wnt signaling driven MSC osteogenic potential.

### Elevated ROS levels contribute to MSC functional defects

**Fig. 6:**
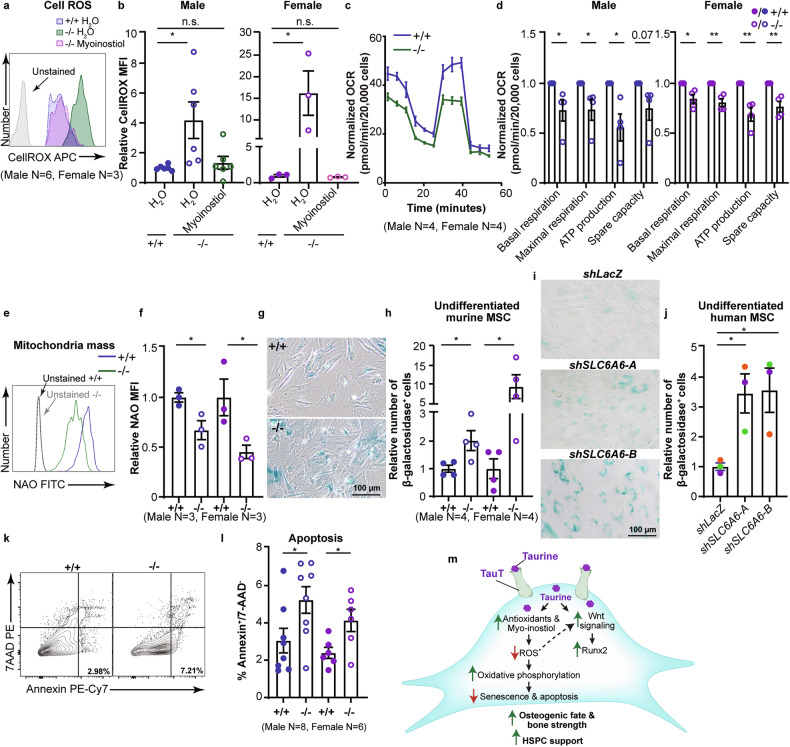
TauT loss promotes oxidative stress and MSC senescence. **a**, **b** Representative FACS histogram (**a**) and relative ROS mean fluorescence intensity (MFI) of male and female (**b**) MSCs treated with myo-inositol (mean ±s.e.m.; data are combined from four independent experiments; one-way ANOVA). **c**, **d** Representative curve of normalized oxygen consumption rate (OCR) in +/+ and −/− MSCs (**c**), normalized OCR quantification in male and female (**d**) MSCs (mean ± s.e.m.; *n* = four-five independent culture wells per cohort; data combined from four independent experiments). **e**, **f** Representative Nonyl Acridine Orange (NAO) FACS histogram (**e**) and quantification of MFI in +/+ and −/− MSCs (**f**) (mean ±s.e.m.; data are combined from two independent experiments). **g**, **h** Microscopy images of senescence-associated β-galactosidase staining (SA-β-gal) (blue) (**g**) and quantification (**h**) of SA-β-gal in undifferentiated murine MSCs (mean ± s.e.m.; data combined from three independent experiments). **i**, **j** Microscopy image of SA-β-gal (blue) (**i**) and quantification (**j**) of SA-β-gal in undifferentiated primary human donor bone marrow-derived MSCs transduced with shRNAs targeting *SLC6A6* or *LacZ* (control) (mean ±s.e.m.; data are combined from three independent experiments with three independent primary samples; one-way ANOVA). **k**, **l** Representative FACS plots (**k**) and quantification of apoptosis (**l**) in +/+ and −/− MSCs (mean ±s.e.m.; data are combined from four independent experiments). **m** Schematic of mechanism. (*p < 0.05, **p < 0.01. ***p < 0.001). All analyses are from unpaired two-tailed Student’s t-test or as indicated.

Taurine and myo-inositol can function as antioxidants and protect cells against oxidative stress induced cell damage [44–46]. We thus determined if TauT loss can affect ROS levels in MSCs. Consistent with the downregulation of antioxidants and upregulation of pathways associated with immune response in TauT^−/−^ MSCs (Fig. 5b, c, S5c), we noted a 4 to 15-fold increase in ROS levels in TauT^−/−^ MSCs compared to controls (Fig. 6a, b). Importantly, myo-inositol supplements could rescue these defects (Fig. 6a, b), indicating that myo-inositol plays a key role downstream of taurine in regulating MSC ROS levels.

Since elevated ROS levels can lead to oxidative-stress mediated cell damage, senescence, and cell death we determined the impact of TauT loss on all these parameters. Our experiments showed that absence of TauT results in 15–20% decrease in basal respiration, 19–27% lower maximal respiration, ~24% reduction in spare capacity, and a 31-44% decrease in ATP production as compared to controls (Fig. 6c, d). However, we did not identify major differences in glycolysis with TauT loss (Fig. S6a, b). Consistent with reduced oxidative phosphorylation, we noted down regulation of the Complex III gene *Mt-cyb* (Fig. S6c), and 33-54% reduced mitochondrial mass (Fig. 6e, f) in TauT^−/−^ MSCs relative to controls.

The elevated ROS levels and defects in oxidative phosphorylation in TauT^−/−^ MSCs were associated with a 2.4-fold increase in cellular senescence in murine TauT^−/−^ MSCs as measured by senescence-associated β-galactosidase (SA-β-gal) staining compared to controls (Fig. 6g, h). Importantly, we also identified a ~3.5-fold increase in SA-β-gal staining in three independent primary human donor bone marrow derived MSCs expressing shRNAs against *SLC6A6* as compared to controls (Fig. 6i, j). Although TauT loss did not affect cell cycle (Fig. S6d, e), it resulted in a 2-fold increase in apoptosis relative to controls (Fig. 6k, l).

Collectively, our data indicates that loss of taurine uptake increases oxidative stress and promotes MSC senescence and cell death (Fig. 6m).

## Discussion

Our work identifies the taurine transporter Slc6a6 as a key regulator of MSC maintenance and function. Our scRNA-seq analysis of murine BMME data sets and in vitro differentiation assays indicate that Slc6a6 expression is largely restricted to MSCs and is rapidly downregulated with differentiation. We do not detect expression of other known taurine transporters in our analysis. These data suggest that TauT expression is restricted to immature MSC populations in murine bone and bone marrow cells, and that TauT is likely the primary source of taurine in MSCs.

MSCs play a critical role in the continuous renewal of bone [22]. Notably, MSCs from juvenile mice (under 8 weeks of age) generally exhibit a faster regenerative capacity compared to adult mice [47]. It is possible that the more pronounced phenotype observed in our studies with 16 week and older mice results from a decline in MSC regenerative potential with consequent effect on physical and mechanical bone properties. Given the minimal impact of taurine loss on femur bone mineral density in mice younger than 16 weeks old, it is possible that other signaling molecules involved in proliferation and differentiation of skeletal system components such as TGF-β and FGF [8, 48] are more crucial during juvenile stages. The mice used in the scRNA-seq datasets analyzed here were 6-22 weeks of age [4, 24, 26]. Thus, assessing Slc6a6 expression during fetal and neo-natal developmental stages may be important to fully understand its influence on bone development. In addition, generating and analyzing Slc6a6 floxed mice may be of value in defining the temporal requirement of Slc6a6 expression in MSCs on bone development, maintenance, and fracture repair.

The observed differences in BMD between males and females may be due to distinct hormonal profiles [49]. Since Slc6a6 is highly expressed in ovarian cells, it is possible that our global Slc6a6 null mice may have impaired ovarian function [50, 51]. As ovaries are the primary source of estrogen, and estrogen is known to regulate bone strength, it is possible that differences in estrogen levels may be contributing to the observed variations in BMD between male and female mice [52]. Thus, future studies on the impact of TauT loss on ovarian function and estrogen levels may help determine the role of estrogen and taurine crosstalk on bone health.

Our data showing that TauT loss does not impair murine muscle morphology or function suggests that the impact of TauT loss on bone is probably not a consequence of declining muscle function at 16 weeks. In addition, we did not find any difference in TRAP expressing osteoclasts with TauT loss in young or middle-aged mice, or any changes in expression of key osteoclast regulatory genes *Rankl* and *Opg* (Fig. S5h). These data suggest that the osteopenia-like phenotype observed with TauT loss likely results from impaired MSC function, and not increased bone resorption. It is also possible that TauT loss impacts bone mineral composition, which can be measured by Raman spectroscopy [53] in future studies.

Our observed in vivo impact of TauT loss on bone strength appears to be less pronounced than the striking effect on MSC function in vitro. This could be due to the activation of compensatory mechanisms in vivo. Consistent with this, our metabolomic analysis shows upregulation of l-ornithine and proline that can drive anti-inflammatory responses, and thus support bone homeostasis [54, 55].

Mechanistically, we find that TauT loss leads to impaired Wnt pathway activation. It is well established that Wnt signaling plays a critical role in regulating MSC osteogenic fate, partly through the upregulation of Runx2 [41, 56]. Consistent with a functional role of Wnt and Runx2 downstream of taurine, we show that GSK3β inhibition can fully rescue osteogenic differentiation in TauT^−/−^ MSCs. In addition, our metabolomic analysis of TauT^−/−^ MSCs identifies a significant downregulation of myo-inositol, a naturally occurring carbohydrate, and a precursor for inositol phosphates [57]. Inositols are known to promote cell signaling and proliferation, antioxidant and metabolic regulation, and Wnt signaling [58, 59]. Consistent with this, myo-inositol not only restored normal ROS levels in TauT^−/−^ MSCs, but also rescued their osteogenic differentiation capacity. Thus, loss of antioxidants like taurine and myo-inositol, and subsequent increase in ROS levels, possibly lead to impaired Wnt activation, senescence, and impaired MSC osteogenic fate (Fig. 6m) [60–62]. Collectively, our data shows that TauT is a key regulator of MSC function, and suggests that modulating taurine uptake in these cells may be of therapeutic interest in instances of bone fracture, osteopenia and osteoporosis.

Patients with acute myeloid leukemia (AML), or those undergoing chemotherapy for solid tumors often develop osteopenia/osteoporosis and reduced bone density [63, 64]. Our recent work has identified that taurine is essential for AML cell growth [4]. In addition, Slc6a6 expression in MSCs decreases during leukemia progression [4]. It is thus possible that rapidly proliferating cancer cells deplete taurine levels in the bone marrow. This may then reduce taurine available for MSCs, thereby promoting osteogenic defects. Given the dual requirement for taurine by leukemia cells as well as MSCs in the bone marrow, it may be of interest to study the dynamics of taurine uptake, and the impact of taurine inhibitors or supplemental taurine, on bone homeostasis in models of these aggressive leukemias.

## Methods

### Analysis of single-cell RNA-sequencing datasets

Data from GEO accessions GSE226644, GSE108892, GSE122467, and GSE128423 was merged and integrated using Seurat v4.1.0 and harmony v0.1.0 within R v4.1.1. Clusters were generated using a resolution of 0.2, marker genes were determined using only positively significantly differentially expressed genes via the FindAllMarkers function, and putative cell types assigned via EnrichR v3.0 against the Azimuth_Cell_Types_2021 database. Clusters expressing CD45, CD71, and Ter119 or annotated as platelets, cycling, or microglia were removed, and this process was repeated.

### Experimental murine model

The *Slc6a6* (TauT) mice were bred as described earlier and are of C57BL/6J background [32]. All mice were 4–40 weeks of age and TauT^+/+^ mice were used as controls. C57BL/6J were obtained from Jackson Laboratory. Mice were bred and maintained in the animal care facilities at the University of Rochester. All animal experiments were performed according to protocols approved by the University of Rochester’s Committee on Animal Resources. All animals used were randomly selected to receive either control or treatments. Animals were selected based on genotype without additional blinding. Age, sex, and environment were controlled for.

### Murine MSC isolation, osteogenic, and adipogenic differentiation

Primary bone marrow cells were harvested from femurs and tibia bone marrow from TauT^+/+^ and TauT^−/−^ mice as previously described [4, 65]. MSCs were plated at a density of 20 × 10^6^  cells per 10 cm dish coated with 5ug/cm^2^ Type I collagen (Corning). Cells were cultured in MEM α without ascorbic acid (Gibco) supplemented with 15% FBS and 100 IU/mL Penicillin/Streptomycin (Gibco) and incubated at 37 °C, 5% CO_2_, and 2% O_2_. Media was changed for 3–5 days to remove non-adherent hematopoietic populations. Once cells reached confluency (7–10 days), MSCs were enriched by magnetic depletion (CD45^-^Ter119^−^CD31^−^) or by flow sorting. Purified MSCs were plated at a density of 3-20 × 10^3^ cells/cm^2^ in a 10 cm dish coated with 5ug/cm^2^ Type I collagen (Corning). Cells were osteo-induced at confluency in their media consisting of MEM α (Gibco) supplemented with 10% FBS, 100 IU/mL Penicillin/Streptomycin (Gibco), 50 mg/ml ascorbic acid (Sigma-Aldrich), and 2.5 mM β-glycerolphosphate (Sigma-Aldrich) for 14 days. Cells were adipo-induced at confluency in their media consisting of MEM α without ascorbic acid (Gibco) supplemented 10% FBS, 100 IU/mL Penicillin/Streptomycin (Gibco), 1 µM Dexamethasone (Cayman Chemical), 0.5 mM IBMX (Sigma), 10 µg/ml Insulin (Humulin) and 1 µM Rosiglidazone (Cayman Chemical). Cells were incubated in induction media for 2 days, then changed to maintenance media consisting of MEM α without ascorbic acid (Gibco) supplemented 10% FBS, 100 IU/mL Penicillin/Streptomycin (Gibco), 10 µg/ml Insulin (Humulin) and 1 µM Rosiglidazone (Cayman Chemical) for 7 days. Cells were stained for mineralization using Alizarin Red S or alkaline phosphatase and von Kossa and quantified as previously described [66, 67]. Cells were stained for lipid formation using Oil Red O and quantified as previously described [67].

### RNA extraction and qRT-PCR

RNA was extracted using the RNeasy Micro kit (Qiagen) as per the manufacturer’s protocols. RNA concentrations were determined using NanoDrop 1000 Spectrophotometer (Thermofisher Scientific). RNA quality was assessed with the Agilent Bioanalyzer 2100 (Agilent Technologies). qRT-PCR was carried out on BioRad CFX96 C100 Thermocycler using BioRad CFX Manager 1.1 v4.1 (BioRad) or Thermofisher Scientific Quant Studio 12 K Flex Real Time PCR using Quant Studio v1.2 (Thermofisher Scientific). qRT-PCR data was analyzed using BioRad CFX Manager 1.1 v4.1 or Quant Studio v1.2.

### Stromal cell isolation and in vivo FACS analysis

Microenvironmental populations were isolated as previously described [4, 68]. Briefly, bone and bone marrow (BM) cells were isolated from long bones in 1x HBSS (Gibco) with 5% FBS (GeminiBio) and 0.5 M EDTA (Gibco). BM cells were digested for 30 min in 1x HBSS containing 2 mg/mL Dispase II (Gibco), 1 mg/mL Collagenase Type IV (Sigma-Aldrich), and 20 ng/mL DNase Type II (Sigma-Aldrich). Bone spicules were digested for 45 min in 1x PBS supplemented with 2.5 mg/mL Collagenase Type I (Stem Cell Technologies) and 20% FBS. Digested BM was RBC lysed using RBC Lysis Buffer (eBioscience). Bone and BM cells were pooled and CD45^+^Ter119^+^ hematopoietic cells were magnetically depleted on an autoMACS cell separator (Miltenyi Biotec). The CD45^−^Ter119^−^ stromal cells were stained and analyzed for candidate populations by flow cytometry on LSRFortessa (BD Biosciences). Antibodies used for defining stromal cell populations were as follows: CD45^−^Ter119^−^CD31^+^Sca1^+^ (arteriolar endothelial cells), CD45^−^Ter119^−^CD31^+^Sca1^−^ (sinusoidal endothelial cells), CD45^-^Ter119^−^CD31^−^Sca1^+^CD51^+^ (MSCs). A detailed list of antibodies is provided in Supplementary Table 1. Data was analyzed using FlowJo software.

### Fibroblast (CFU-F) and osteoblast (CFU-OB) colony formation unit assays

Bone marrow cells were isolated from mice as previously described [69]. Briefly, bone marrow cells were seeded at a density of 25,000 cells/cm^2^. Media was exchanged for 3 days to remove non-adherent hematopoietic populations. Osteoinduction was performed as described above. For assessment of CFU-F, cells were cultured in MEM α without ascorbic acid (Gibco) supplemented with 15% FBS (GeminiBio) and 100 IU/mL Penicillin/Streptomycin (Gibco). CFU-OB and CFU-F were incubated at 37 °C, 5% CO_2_, and 2% O_2_ for 14 days. Myo-inositol (I5125, Sigma-Aldrich) and CHIR99021 (HY-10182, MedChemExpress) were used as described in results. On day 14, cells were either stained with crystal violet (CFU-F) or alkaline phosphatase and von Kossa (CFU-OB) to assess total colonies. Clusters of greater than 50 cells were considered a CFU. Plates were imaged on a LI-COR Odyssey M. Cells were quantified using ImageJ.

### Primary human donor bone marrow derived MSC osteogenic and adipogenic differentiation

Primary human donor bone marrow MSCs were cultured from marrow aspirates from healthy donors, obtained after written informed consent in accordance with the Declaration of Helsinki and approval of University of Rochester institutional review board (IRB). Primary human donor bone marrow MSCs were differentiated as described above for murine cells. All shRNA sequences are as described earlier [4].

### Coculture of KLS cells and MSCs

MSCs were isolated from +/+ and −/− mice as described above. 100,000 MSCs per well were plated in a 24-well plate. After 24 hours, 20,000 C57BL/6J KLS cells (Lin^−^cKit^+^Sca1^+^) were FACS sorted and cocultured with MSCs in RPMI (Gibco) media supplemented with 10% Hi-FBS (GeminiBio), 50 mM 2-mercatpoethanol, and IU/mL Penicillin-Streptomycin (Gibco), media adapted from earlier work [70]. Post 72 hours, the hematopoietic suspended cells were removed for viability analysis, flow cytometry, and CFU assays. Viability was assessed using 0.4% Trypan blue (Thermofisher) on an Olympus CK2 microscope. Cell lineage content was assessed via flow cytometry on a LSRFortessa (BD Biosciences). Antibodies used for defining lineage^+^ are as followed: CD3ε, CD4, CD8, Gr1, CD11b/Mac-1, Ter119, CD45R/B220 and CD19. Data was analyzed using FlowJo software. Colony forming ability was assessed by plating 400 cells in methylcellulose medium (M3434, StemCell Technologies). Colonies were scored on day 7 using an Olympus CK2 microscope. Clusters of greater than 50 cells were considered a CFU.

### Dual-energy X-ray absorptiometry (DEXA)

Male and female +/+ and −/− mice were measured between 4 and 40 weeks old. Mice were weighed, then anesthetized using either 100 mg/kg Ketamine and 10 mg/kg Xylazine IP at 0.1 mL per 10 grams of body weight or 2% isoflurane vapor. Under anesthesia, the mouse’s whole body was scanned, and an area of interest was selected using Lunar PIXImus2 system. The whole body and femur were analyzed in terms of bone mineral density (BMD) and fat percentage.

### Bone micro-computed tomography (micro-CT)

TauT +/+ and −/− femurs were isolated, cleaned of excess soft tissue, and stored in 1x PBS at −80 °C prior to micro-CT. Specimens were imaged using high-resolution acquisition (10.5 µm voxel size) with the VivaCT 40 tomograph (Scanco Medical). Scanco analysis software was utilized for volume quantification.

### Biomechanical torsional testing

TauT +/+ and −/− femurs were isolated, cleaned of excess soft tissue, and stored in 1x PBS at −80 °C prior to biomechanical testing. Tibia samples were held in bone cement and tested using EnduraTec TestBench system (Bose). The tibiae were tested in torsion until failure at a rate of 1°/s. The torque data were plotted against rotational deformation to determine maximum torque and torsional rigidity.

### Grip strength test

Grip strength was analyzed for limbs using a digital force meter (Columbus Instruments Grip Strength Meter) equipped with precision force gauges to retain the peak force applied. Each mouse was tested 5 times with a minimum 60 second inter-trial interval.

### Histological staining

TauT +/+ and −/− femurs were isolated, cleaned of excess soft tissue, and were fixed for three days in 10% neutral buffered formalin (NBF) solution and processed for histology using decalcification in Webb-Jee 14% EDTA solution for two weeks followed by paraffin embedding. Samples were sectioned at 5 µm and stained with tartrate-resistant acid phosphatase (TRAP). +/+ and −/− gastrocnemius muscles were isolated, cleaned of excess soft tissue, and fixed for two days in 10% NBF solution and processed for histology. Samples were sectioned at 5 µm and stained with hematoxylin and eosin (H&E). Microscopy images were obtained on an Olympus CKX41 microscope using CellSens Entry v2.3 (Olympus). The number of TRAP positive cells and myofiber cross-sectional area was measured in ImageJ.

### Sample preparation for untargeted metabolomics of murine TauT^+/+^ and TauT^−/−^ MSCs

TauT +/+ and −/− MSC cultures were washed with PBS and detached using TrypLE. Cells were washed with PBS containing 5 mM glucose, centrifuged at 3000x g for 1 min, and snap frozen. Frozen cell pellets were resuspended at 1 million cells per 1 ml of 40:40:20 MeOH:ACN:H_2_0 via vortexing, transferred to −20 °C for 30 min and then ice for 30 min with vortexing every 10 min. Next, samples were centrifuged at 17,000x g for 10 min and 90% of supernatant was dried down in a vacuum evaporator (Thermo). Samples were reconstituted in 50% acetonitrile (A955, Fisher Scientific), at a volume equal to 10% of dried down volume and transferred to glass vials for LC/MS analysis.

### LC/MS analysis

For LC-MS analysis, metabolite extracts were analyzed by high resolution mass spectrometry with an Orbitrap Exploris 240 (Thermo) coupled to a Vanquish Flex liquid chromatography system (Thermo). 2 µL of each sample was injected on a Waters XBridge XP BEH Amide column (150 mm length × 2.1 mm id, 2.5 µm particle size) maintained at 25 °C, with a Waters XBridge XP VanGuard BEH Amide (5 mm × 2.1 mm id, 2.5 µm particle size) guard column. For positive mode acquisition, mobile phase A was 100% LC-MS grade H2O with 10 mM ammonium formate and 0.125% formic acid. Mobile phase B was 90% acetonitrile with 10 mM ammonium formate and 0.125% formic acid. For negative mode acquisition, mobile phase A was 100% LC-MS grade H_2_O with 10 mM ammonium acetate, 0.1% ammonium hydroxide, and 0.1% medronic acid (Agilent). Mobile phase B was 90% acetonitrile with 10 mM ammonium acetate, 0.1% ammonium hydroxide, and 0.1% medronic acid. The gradient was 0 min, 100% B; 2 min, 100% B; 3 min, 90% B; 5 min, 90% B; 6 min, 85% B; 7 min, 85% B; 8 min, 75% B; 9 min, 75% B; 10 min, 55% B; 12 min, 55% B; 13 min, 35%, 20 min, 35% B; 20.1 min, 35% B; 20.6 min, 100% B; 22.2 min, 100% B all at a flow rate of 150 μl/min, followed by 22.7 min, 100% B; 27.9 min, 100% B at a flow rate of 300 μl/min, and finally 28 min, 100% B at flow rate of 150 μl/min, for a total length of 28 min. The H-ESI source was operated in positive mode at spray voltage 3500 or negative mode at spray voltage 2500 with the following parameters: sheath gas 35 au, aux gas 7 au, sweep gas 0 au, ion transfer tube temperature 320 °C, vaporizer temperature 275 °C, mass range 70 to 1000 m/z, full scan MS1 mass resolution of 120,000 FWHM, RF lens at 70%, and standard automatic gain control (AGC). Data dependent MS2 (ddMS2) fragmentation for compound identification and annotation was performed via the AquireX workflow (Thermo Scientific) comprised of three deep scans with MS1 resolution at 60,000 and MS2 resolution at 15,000. LC-MS data were analyzed by Compound Discover (v3.3, Thermo Scientific) and El-Maven software (ref below) for peak area determination and compound annotation. Compounds were annotated by matching to LC-MS method specific retention time values of external standards and MS2 spectral matching to external standards and the mzCloud database (Thermo Scientific).

For statistical analysis, metabolite values were auto scaled and raw *p*-values were calculated via pairwise Student’s t-tests between the indicated sample groups. Adjusted *p*-values were computed using the Benjamini-Hochberg False Discovery Rate (FDR) correction on the raw *p*-values.

### Bulk RNA-sequencing of TauT^+/+^ and TauT^−/−^ MSCs

1 ng of total RNA was used as input in the SMART-Seq mRNA LP (Takara Bio USA, San Jose, CA) library prep workflow per manufacturer’s recommendations. Briefly, full-length cDNA was synthesized via oligo-dT priming and template-switching at the 5’ end. cDNA libraries were then amplified by long-distance PCR. The quantity and quality of the amplified cDNA was determined using the Qubit Fluorometer (Life Technologies, Carlsbad, CA) and the Agilent Bioanalyzer 2100 (Santa Clara, CA). Subsequently, 1 ng of amplified cDNA was used for library construction through enzymatic fragmentation and ligation of stem-loop adapters, which provide binding sites for final library amplification. Lastly, Illumina-compatible libraries were amplified using unique dual-indexed primers. Amplified libraries were assessed for quantity and quality using the Qubit Fluorometer and the Agilent Fragment Analyzer and prepared to sequence on the Illumina NovaSeq X Plus (Illumina, San Diego, CA) 10B flowcell with paired-end reads of 150nt.

Raw reads generated from the Illumina basecalls were demultiplexed using bcl-convert v4.1.7. Quality filtering and adapter removal was performed using FastP v.0.23.1. Processed reads were then mapped to the human reference genome (GRCm39 + gencode M31) (https://www.gencodegenes.org/mouse/release_M31.html) using STAR_2.7.9a. Reads mapping to genes were counted using subread featurecounts v2.0.1 with “-s 2”. Differential expression analysis was performed using DESeq2-1.34.0 with an adjusted *P*-value threshold of 0.05 within R v4.1.1 A design formula incorporating both sex and condition was used to regress out the impacts of sex in differential expression. Gene ontology analyses were performed using the EnrichR-3.0 package.

### Western blot analysis

MSCs were isolated from +/+ and −/− mice as described above. Cell lysates were prepared in 1x RIPA (Thermo Scientific) supplemented with 1x protease/phosphatase inhibitors (Cell Signaling Technology, CST) and 250 IU Benzonase Nuclease (Millipore Sigma). Samples were separated on gradient polyacrylamide gels and transferred to nitrocellulose blotting membrane (0.45 µM; GE healthcare). Primary antibodies against non-phosphorylated active beta-catenin (β-catenin), Runx2, Actin, and GAPDH (CST) were used. Horse-radish peroxidase (HRP)-conjugated anti-rabbit antibody (CST) was used to detect primary antibodies. Immunoblots were developed using SuperSignal West Femto Maximum Sensitivity Substrate (Thermo Scientific). Immunoblots were imaged using LI-COR Odyssey M using Empiria Studio v2.3 (LI-COR). Images were analyzed using Empiria Studio v3.2.0.186 (LI-COR).

### Flow cytometry-based analysis of CellROX

MSCs were isolated from +/+ and −/− mice as stated above. Cells were treated with water (control) or Myo-inositol (I5125, Sigma-Aldrich), and ROS levels were analyzed using CellROX Deep Red Reagent (Thermo Fisher Scientific) according to manufacturer’s protocol. Cells were analyzed via FACS on LSRFortessa (BD Biosciences). Mean fluorescence intensity (MFI) of CellROX was quantified using FlowJo.

### Seahorse assays

The Seahorse XF cell Mito Stress Test Kit (Agilent Technologies, 103015-100) was used to measure glycolytic flux (ECAR) and oxygen consumption (OCR) respectively. 20,000 MSCs were seeded in XF96 plates in MEM α without ascorbic acid (Gibco) supplemented with 15% FBS (GeminiBio) and 100 IU/mL Penicillin/Streptomycin (Gibco). Cells were incubated for 24 hours at 37 °C, 5% CO_2_, and 2% O_2_. Following 24 hours, OCR and ECAR data was measured following sequential addition of oligomycin (1.5 µM), carbonyl cyanide-4 (trifluoromethoxy) phenylhydrazone (FCCP) (0.5 µM), rotenone and antimycin (0.5 µM) using XF96 analyzer. Data was analyzed using Wave v2.6.3 (Agilent Technologies).

### Mitochondrial mass assay

MSCs were isolated from +/+ and −/− mice as stated above. Mitochondrial mass analysis was carried out as previously described [66]. Briefly, MSCs were stained with 100 nM of nonyl acridine orange (NAO) (Invitrogen) for 30 min at 37 °C. Cells were analyzed via FACS on LSRFortessa (BD Biosciences). Mean fluorescence intensity of NAO was quantified using FlowJo.

### Senescence associated beta-galactosidase staining of murine and human MSC

Murine and human MSCs were cultured as described above. Senescence associated beta-galactosidase staining was carried out according to manufacturer’s protocol (Cell Signaling Technology). Cells were analyzed using ImageJ.

### Annexin V and bromodeoxyuridine (BrdU) incorporation assays

Apoptosis was measured using annexin V and 7AAD (eBiosciences). In vitro BrdU incorporation was assessed using the FITC BrdU Flow Kit (BD Biosciences) according to manufacturer’s protocol. Data was analyzed using using FlowJo.

### Statistical analysis

Statistical analyses were carried out using Graphpad Prism software v6.0 (GraphPad software Inc.). Data are mean ± SEM or mean ± SD. One-way ANOVA, unpaired two-sided Student’s t-tests, and multiple unpaired t-tests corrected with Benjamin & Hochberg method, were used to determine statistical significance. For statistical analysis of only two groups with parametric conditions, we used an unpaired two-tailed Students t-test. When comparing multiple sets of two groups however, a multiple unpaired t-test was used with the correction method of Benjamin & Hochberg to control type 1 errors. To determine if there was a difference in mean between three or more groups, a one-way analysis of variance (ANOVA) was performed, as only one independent variable was compared amongst the groups. The ANOVA test was followed by Tukey’s post-hoc test for multiple comparisons, to determine which groups were statistically significant. No statistical method was used to predetermine sample size for experiments. Data was collected depending on the nature of the experiments and proper statistical analysis. Adequate sample size was determined based on previous work [4, 66, 67].

## Supplementary information

Figure S1

Figure S2

Figure S3

Figure S4

Figure S5

Figure S6

Figure S7

Supplementary Figure Legends

List of Antibodies

## Data Availability

The murine bulk RNA-sequencing data for this publication is available under GEO accession GSE297037. TauT +/+ and −/− MSC metabolomics data (ST004302) are available online at the Metabolomics Workbench at https://www.metabolomicsworkbench.org/.
