# Supplementary figures and images for: Taurine transporter SLC6A6 expression promotes mesenchymal stromal cell function
Source: Cell Death Dis. 2026 Jan 8;17(1):14. doi: 10.1038/s41419-025-08233-4 (PMC12783680; doi:10.1038/s41419-025-08233-4)

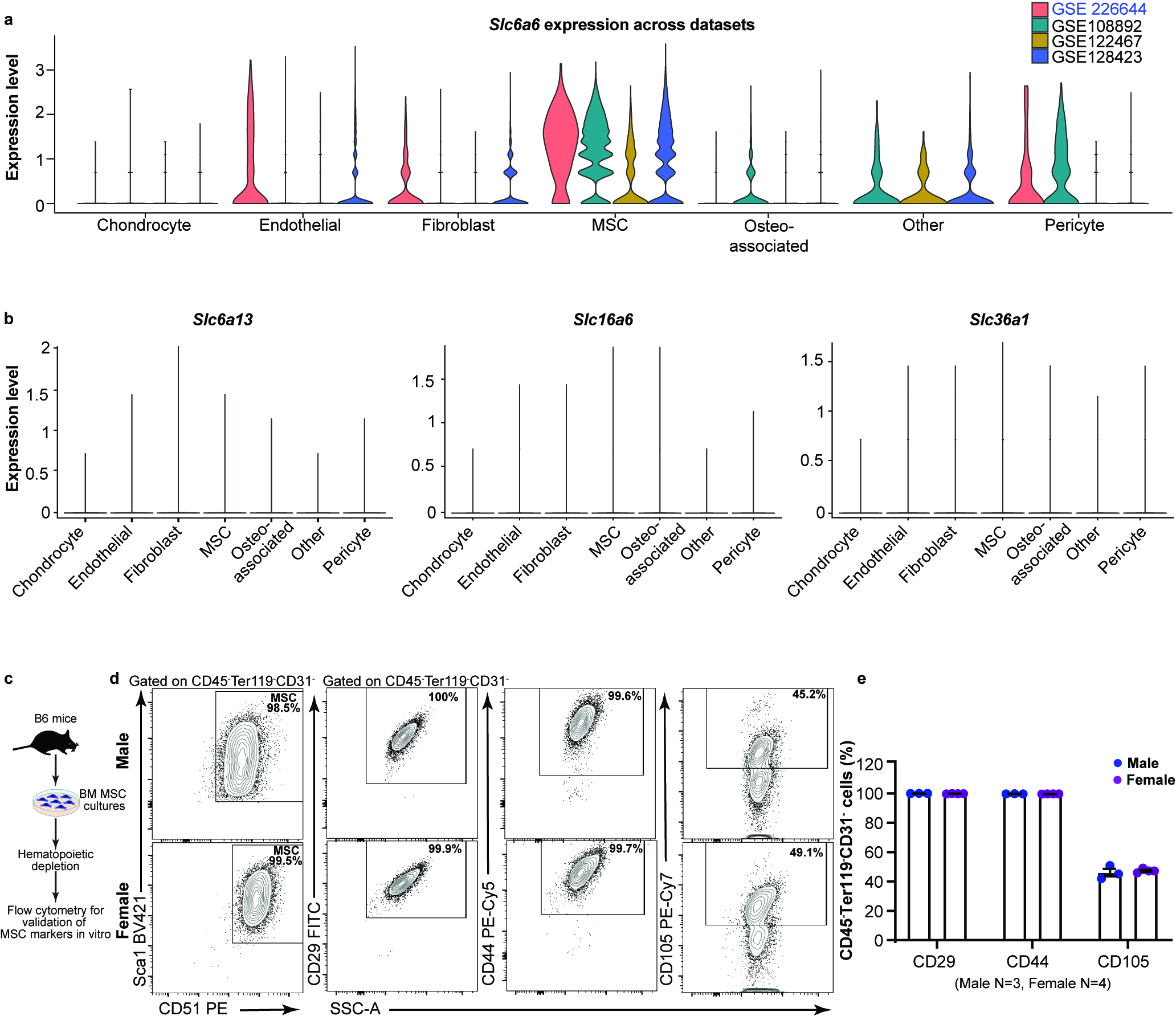

Supplement: Supplementary file 1 — Figure S1 [file 41419_2025_8233_MOESM1_ESM.tif]

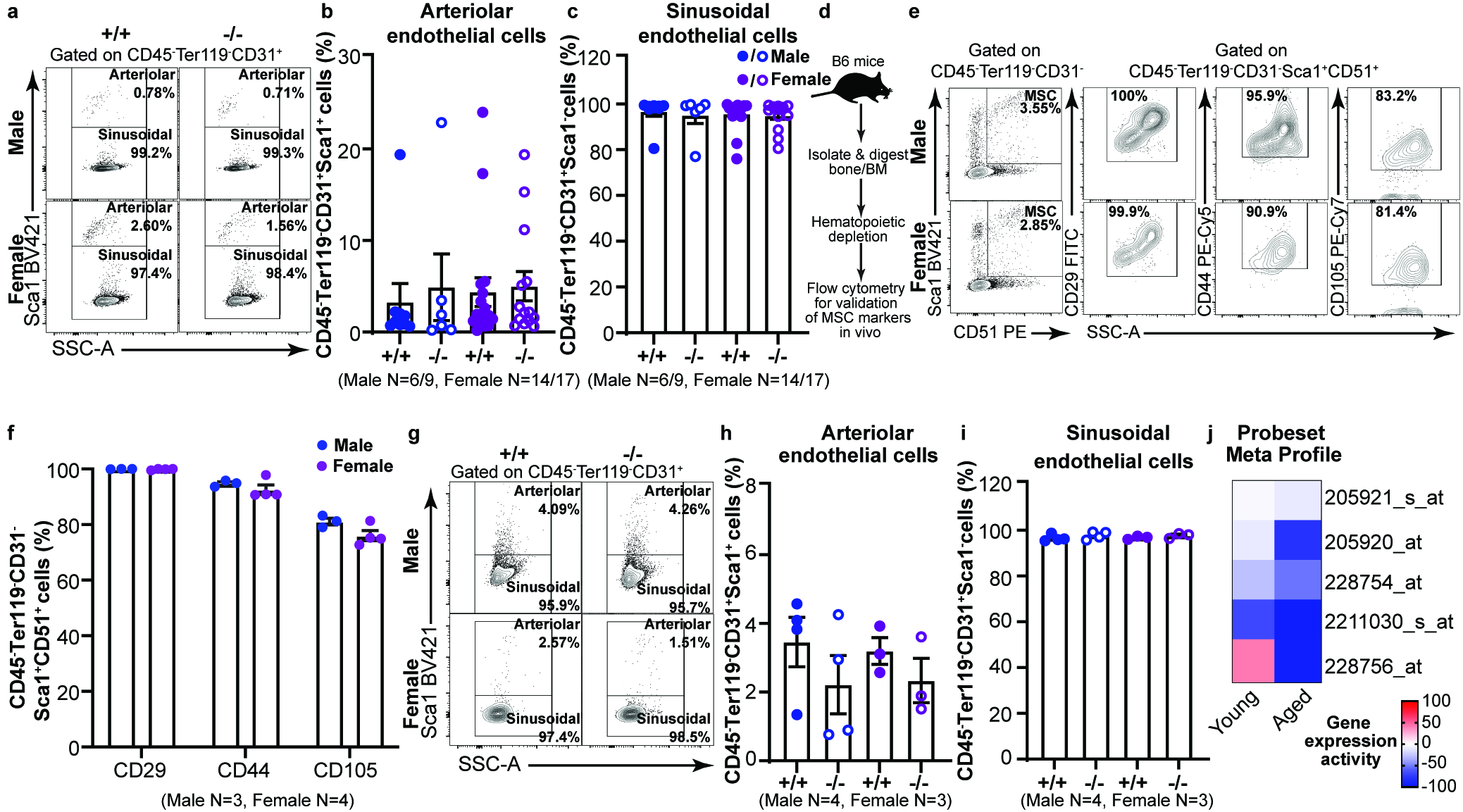

Supplement: Supplementary file 2 — Figure S2 [file 41419_2025_8233_MOESM2_ESM.tif]

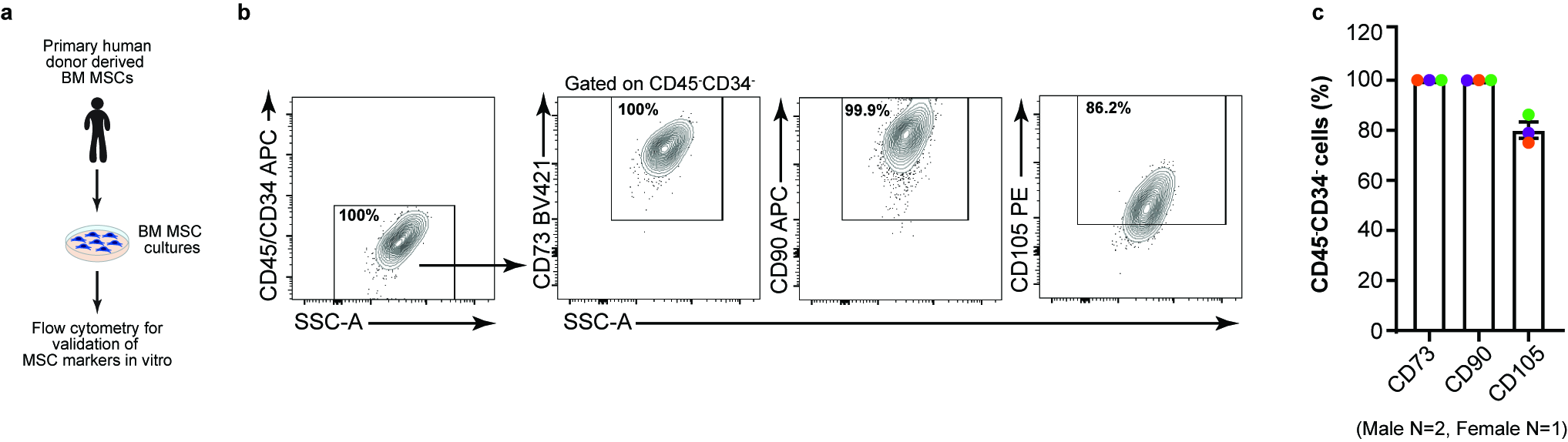

Supplement: Supplementary file 3 — Figure S3 [file 41419_2025_8233_MOESM3_ESM.tif]

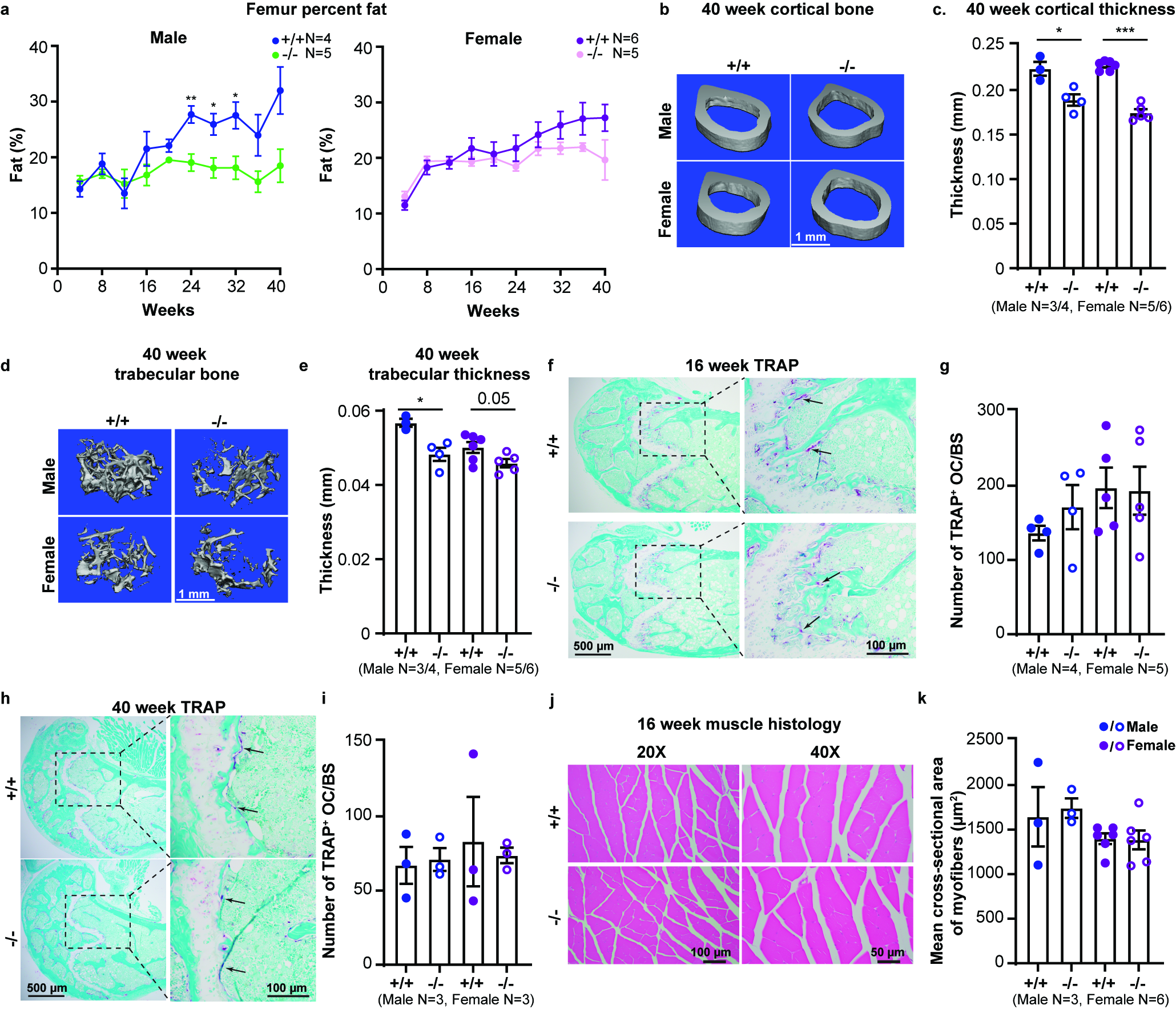

Supplement: Supplementary file 4 — Figure S4 [file 41419_2025_8233_MOESM4_ESM.tif]

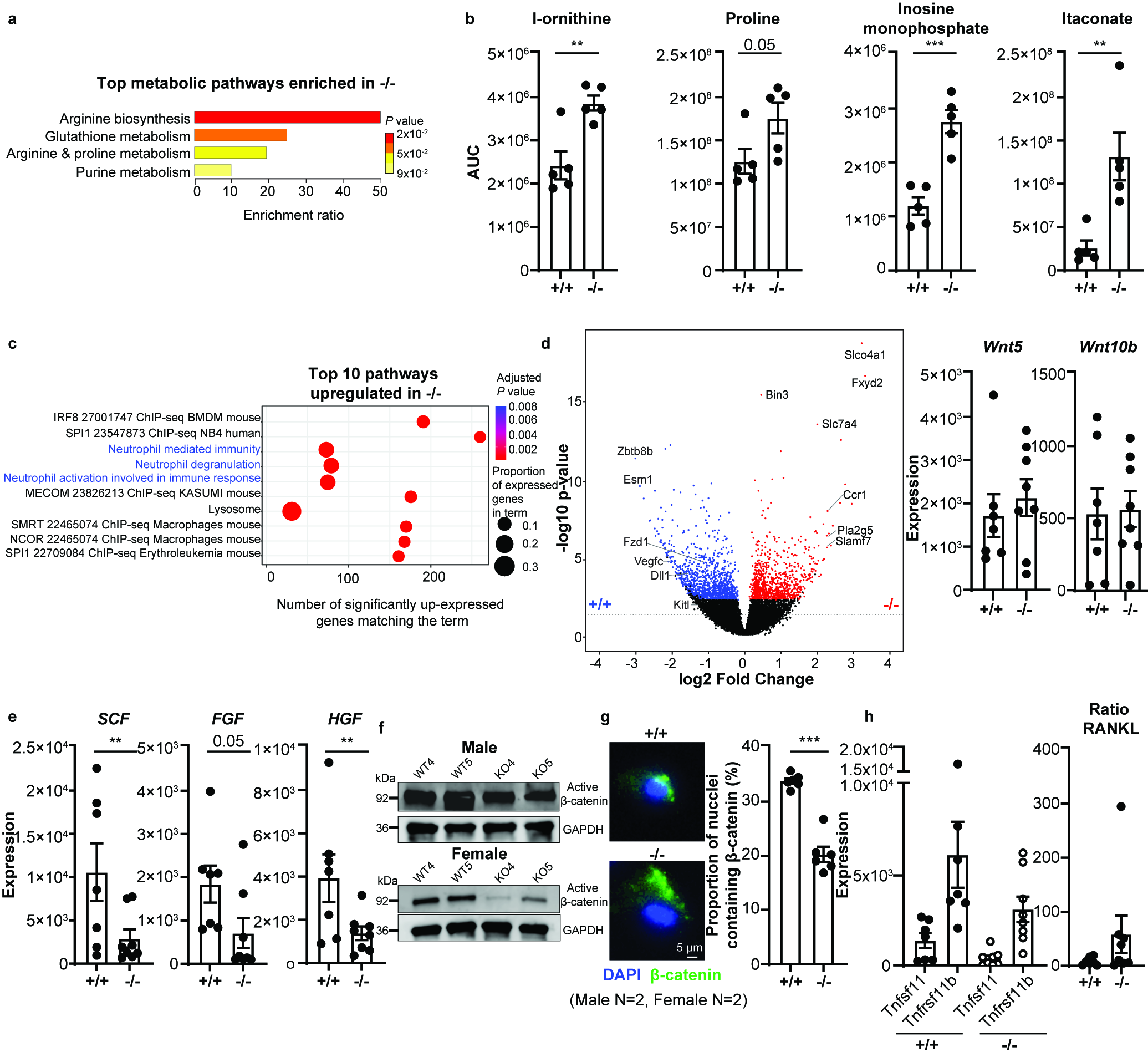

Supplement: Supplementary file 5 — Figure S5 [file 41419_2025_8233_MOESM5_ESM.tif]

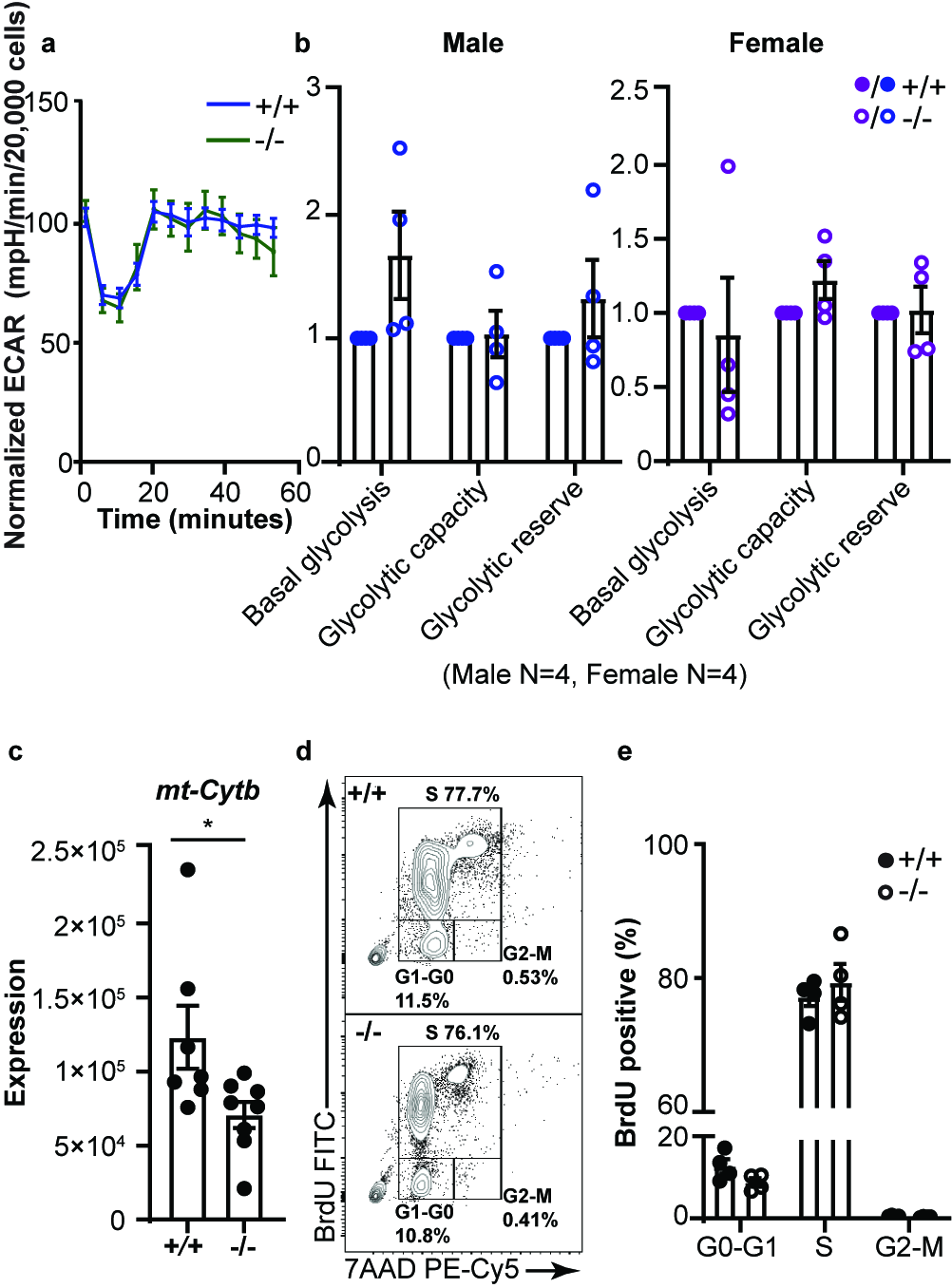

Supplement: Supplementary file 6 — Figure S6 [file 41419_2025_8233_MOESM6_ESM.tif]

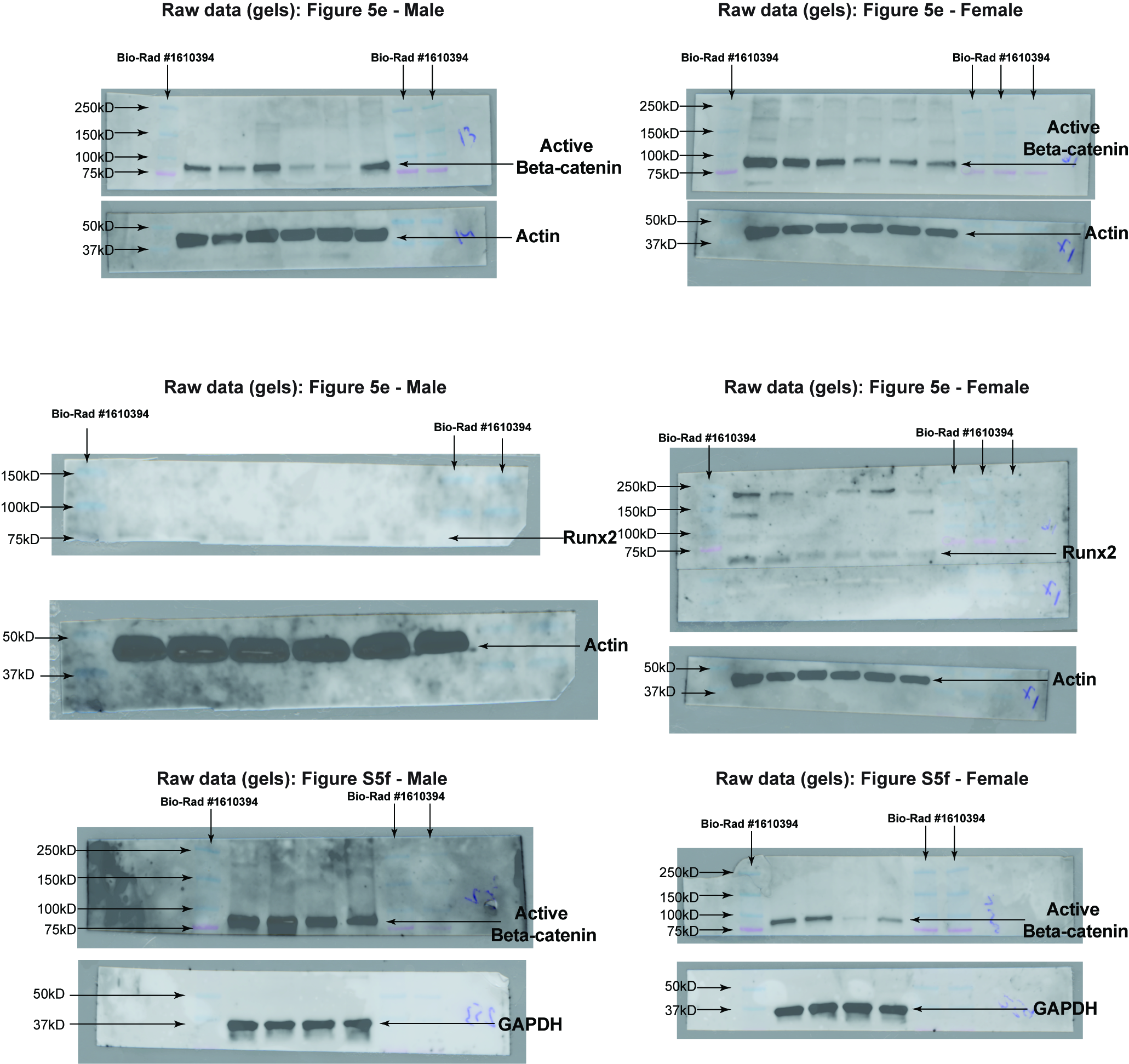

Supplement: Supplementary file 7 — Figure S7 [file 41419_2025_8233_MOESM7_ESM.tif]
