# Supplementary material for: Taurine transporter SLC6A6 expression promotes mesenchymal stromal cell function
Source: Cell Death Dis. 2026 Jan 8;17(1):14. doi: 10.1038/s41419-025-08233-4 (PMC12783680; doi:10.1038/s41419-025-08233-4)
Supplement: Supplementary file 8 — Supplementary Figure Legends [file 41419_2025_8233_MOESM8_ESM.docx]

**SUPPLEMENTARY FIGURE LEGENDS**

**Figure S1: Taurine transporter expression across various data sets.**

**a, b,** Violin plots of Slc6a6 expression (**a**) and Slc6a13, Slc16a6, and Slc36a1 expression (**b**) in non-hematopoietic cells in publicly available single-cell RNA-sequencing data sets. **c,** Experimental strategy used to characterize murine MSC surface markers from *in vitro* cultures. **d**, **e**, Representative FACS plots (**d**), and quantification (**e**) of MSC surface markers in bone marrow stroma (mean ±s.e.m.; data are combined from two independent experiments).

**Figure S2: Characterization of murine mesenchymal stromal cells surface markers *in vivo.***

**a-c**, Representative FACS plots (**a**) and quantification of arteriolar endothelial cells (**b**) and sinusoidal endothelial cells (**c**) in 16 week old bone marrow stroma (mean ± s.e.m.; data are combined from five independent experiments). **d,** Experimental strategy used to characterize murine MSC surface markers from *in vivo* from wild-type mice. **e**, **f**, Representative FACS plots (**e**), and quantification (**f**) of MSC surface markers (mean ±s.e.m.; data are combined from two independent experiments). **g-i**, Representative FACS plots (**g**) and quantification of arteriolar endothelial cells (**h**) and sinusoidal endothelial cells (**i**) in 40 week old bone marrow stroma (mean ± s.e.m.; data are combined from three independent experiments). **j,** SLC6A6 expression in young (26 year old) versus aged (91 year old) hip skeletal stem/progenitor cells from publicly available Microarray data set ^46^. (*p<0.05, **p<0.01. ***p<0.001). All analyses are from unpaired two-tailed Student’s t-test.

**Figure S3: Characterization of primary human donor bone marrow derived mesenchymal stromal cells surface markers *in vitro*.**

**a**, Experimental strategy used to characterize human MSC surface markers *in vitro*. **b**, **c**, Representative FACS plots (**b**), and quantification (**c**) of human MSC surface markers (mean ±s.d.; each color represents an independent human MSC sample).

**Figure S4: MSCs are responsible for observed physical bone defects in the absence of TauT *in vivo.***

**a**, DEXA scans of femur percent fat of TauT^+/+^ (+/+) and TauT^-/-^ (-/-) mice over a 40 week period (mean ±s.e.m.; data are combined from four independent experiments). **b**, **c**, Micro-CT pictographs of 40 week femur cortical region (**b**) and quantification of cortical thickness (**c**) (mean ±s.e.m.; data are combined from three independent experiments). **d, e**, Micro-CT pictographs of 40 week femur trabecular region (**d**) and quantification of trabecular thickness (**e**) (mean ±s.e.m.; data are combined from three independent experiments). **f**, **g**, Representative tartrate resistant acid phosphatase (TRAP) staining of 16 week murine femurs (**f**) and quantification (**g**) of the number of TRAP^+^ osteoclasts/bone surface (mean ±s.e.m.; data are combined from four independent experiments). **h**, **i**, Representative tartrate resistant acid phosphatase (TRAP) staining of 40 week murine femurs (**h**) and quantification (**i**) of the number of TRAP^+^ osteoclasts/bone surface (mean ±s.e.m.; data are combined from three independent experiments). **j**, **k**, Representative Hematoxylin and Eosin (H&E) staining of gastrocnemius muscle in 16 week +/+ and -/- mice (**j**) and quantification of myofiber cross-sectional area (**k**) (mean ±s.e.m.; data are combined from three independent experiments). (*p<0.05, **p<0.01. ***p<0.001). All analyses are from unpaired two-tailed Student’s t-test or as indicated.

**Figure S5: Transcriptomic analysis of MSCs.**

**a**, **b**, Unbiased Enrichr analysis (MetaboAnalyst; Padj ≤ 0.05) of top metabolic pathways enriched in -/- (**a**) and quantification of indicated metabolites from untargeted metabolomics (**b**) (mean ±s.e.m.; data are combined from five independent MSC samples). **c**, Gene sets significantly upregulated in -/- MSCs (blue text indicates pathways of interest). **d**, Volcano plot of genes upregulated in +/+ (blue) and -/- (red) MSCs and expression of Wnt5 and Wnt10b in +/+ and -/- MSCs using our RNA-sequencing data set (n=8 male and n=7 female MSCs). **e**, Expression of SCF, FGF, and HGF in +/+ and -/- MSCs using our RNA-sequencing data set (n=8 male and n=7 female MSCs; mean ±s.e.m.). **f**, immunoblot of indicated proteins (mean ±s.e.m.; data are combined from two independent experiments). **g**, Representative microscopy images and quantification of nuclear β-catenin (green) and DAPI (blue) in +/+ and -/- MSCs (mean ±s.e.m.; data are combined from two different experiments). **h**, Expression and ratio of RANKL gene (*Tnfsf11*) to Opg gene (*Tnfrsf11b*) in +/+ and -/- MSCs using our RNA-sequencing data set (n=8 male and n=7 female MSCs; mean ±s.e.m.). All analyses are from unpaired two-tailed Student’s t-test. (*p<0.05, **p<0.01. ***p<0.001).

**Figure S6: TauT loss does not impact extracellular acidification or MSC proliferation.**

**a, b**, Representative curve of normalized extracellular acidification rate (ECAR) in +/+ and -/- MSCs (**a**), normalized ECAR quantification in male and female (**b**) MSCs (mean ± s.e.m.; n=four-five independent culture wells per cohort; data combined from four independent experiments). **c,** Expression of *mt-Cytb* in +/+ and -/- MSCs using our RNA-sequencing data set (n=8 male and n=7 female MSCs; mean ±s.e.m.). **d,** **e**, Representative FACS plots (**d**), and quantification (**e**) of frequency of *in vitro* BrdU incorporation in +/+ and -/- MSCs (mean ±s.e.m.; n=four independent MSCs; data are combined from two independent experiments). (*p<0.05, **p<0.01. ***p<0.001). All analyses are from unpaired two-tailed Student’s t-test or as indicated.
